# Supplementary material for: Sex-specific impact of B cell-derived IL-10 on tuberculosis resistance
Source: Front Immunol. 2025 Apr 7;16:1524500. doi: 10.3389/fimmu.2025.1524500 (PMC12009811; doi:10.3389/fimmu.2025.1524500)
Supplement: Supplementary file 1 [file DataSheet1.docx]

**Supplementary Figures and Tables**


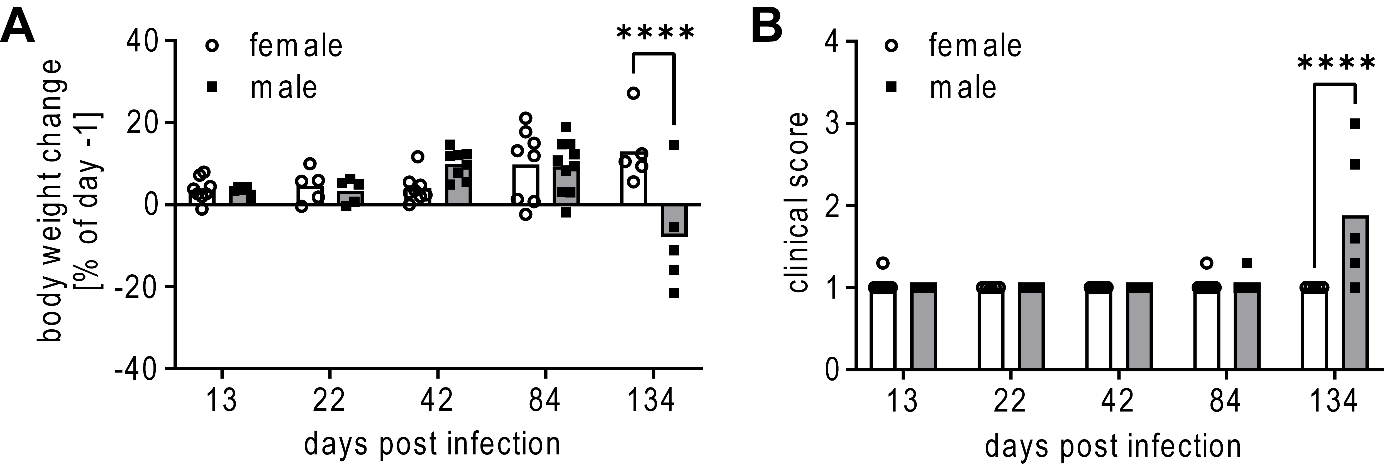


**Figure S1**. *Accelerated disease progression in male Vert-X mice*. Male and female Vert-X mice were infected via the aerosol route with a low dose of *Mtb* HN878. Body weight change in % (A) and clinical scores (B) are shown from n = 5-10 mice per time point. Statistical analysis was performed by 2way ANOVA followed by Tukey's multiple comparisons test. ****p<0.0001


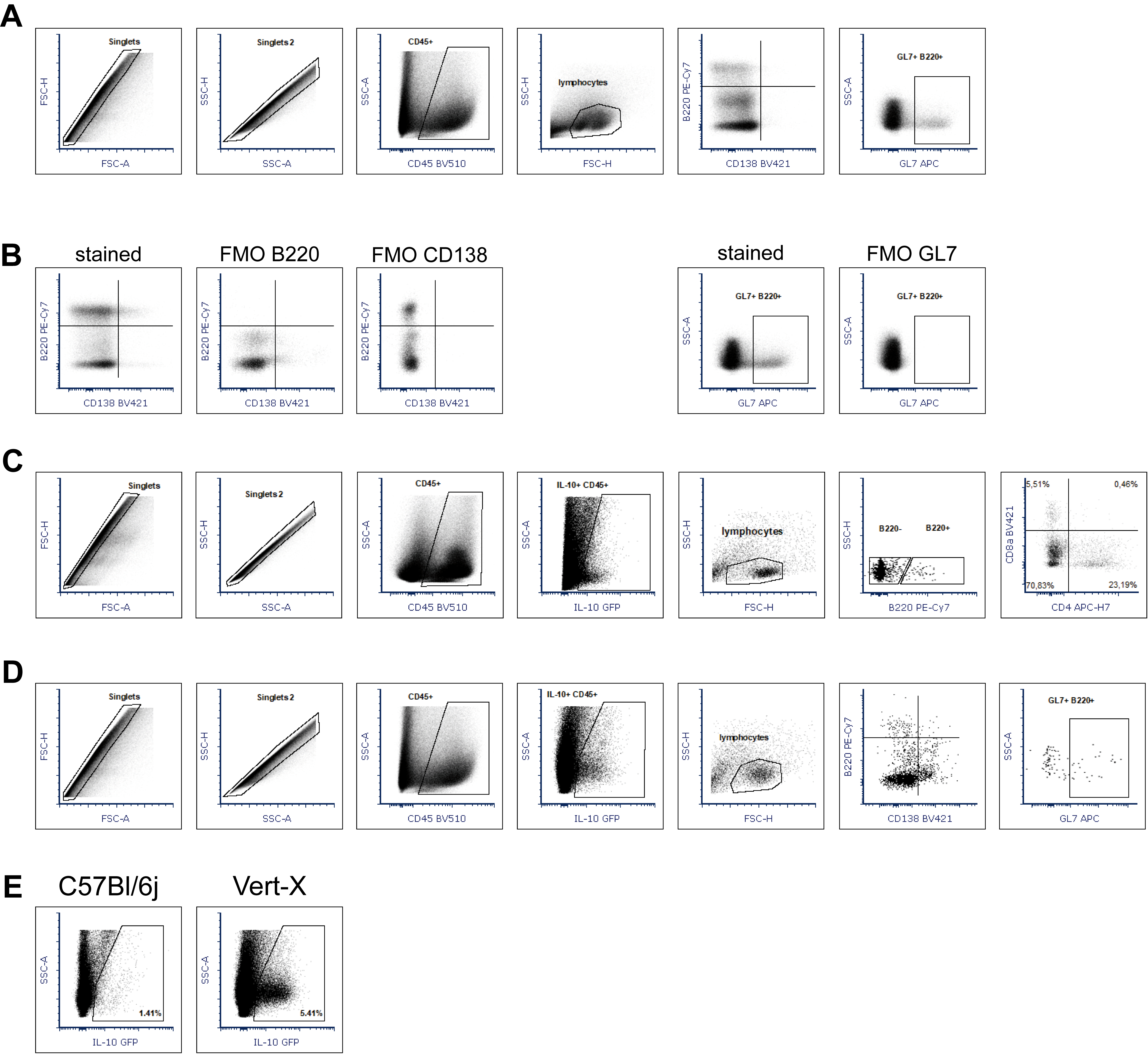


**Figure S2**. *Gating strategy to analyze in vivo IL-10 expression in Mtb infected lungs*. Representative flow cytometry plots for detection of B cell subtypes (A) using FMOs for B220, CD138, and GL7 (B). IL-10 expression in T cells (C) and B cell subtypes (D). An uninfected wild type C57BL/6j mouse was used as negative control for IL-10 expression (E).


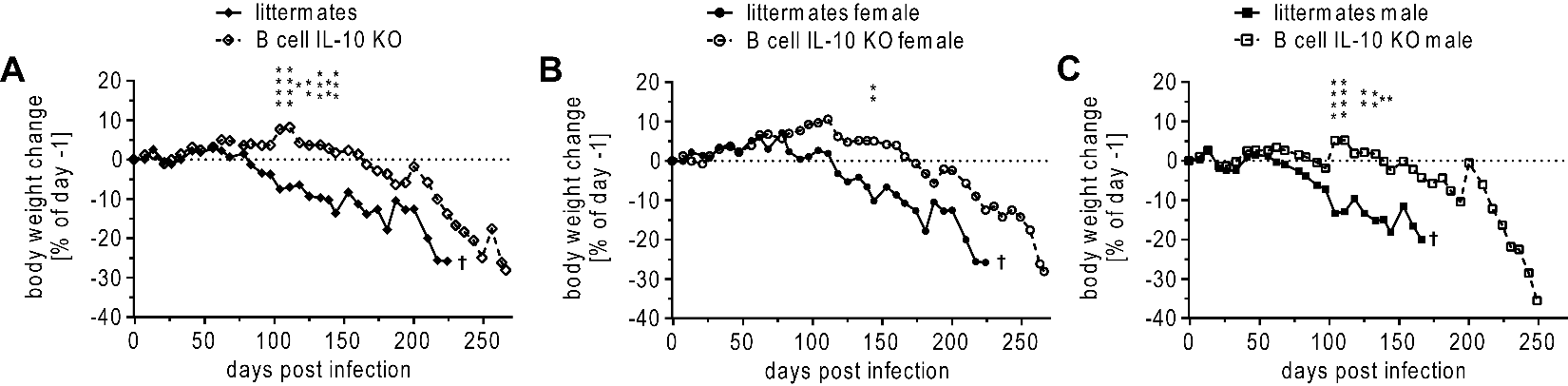


**Figure S3**. *Body weight changes of Mtb HN878 infected B cell IL-10 KO mice and littermates*. A) Body weight change (%) of B cell IL-10 KO mice (n = 18) and B cell IL-10 competent littermates (n = 12). Data stratified by sex for (B) females (n = 9 B cell IL-10 KO; n = 5 littermates) and (C) males (n = 9 B cell IL-10 KO; n = 7 littermates). Data represent the mean body weight change of animals from one experiment. Statistical analysis was performed by 2way ANOVA followed by Tukey's multiple comparisons test. *p<0.05; **p<0.01; ***p<0.001; ****p<0.0001


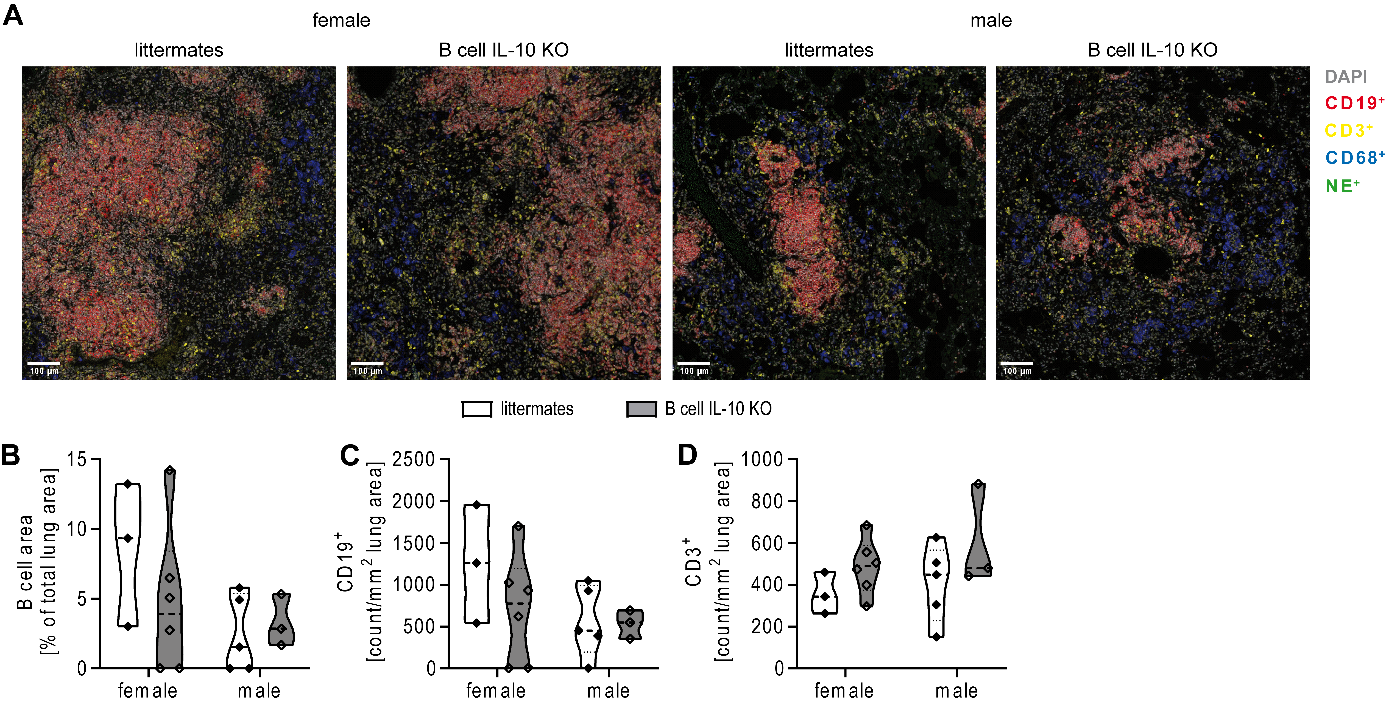
**Figure S4**. *Examination of immune cell populations within the infected lung using mIF.* A) Representative micrographs from lungs of one female and male mouse per group stained with antibodies to detect B cells (CD19^+^), T cells (CD3^+^), neutrophils (NE^+^) and macrophages (CD68^+^); Bar = 100 µm. B-D) Quantitative analysis of B cell area and respective immune cells as shown in (A).

**Table S1.** *Pipetting protocol for mIF staining.* Antibody targets and staining protocol to investigate immune cell populations within the infected lung of female and male B cell IL-10 KO mice and their littermates using mIF.


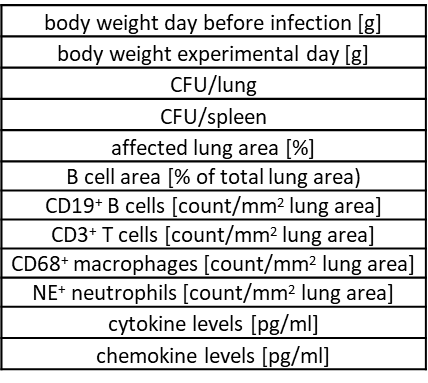


**Table S2.** *List for PCA.* The various data obtained for female (n=6) and male (n=3) B cell IL-10 KO mice and their female (n=3) and male (n=5) B cell IL-10 competent littermates at day 82 after infection were used to generate a PCA plot.
